# Metagenomic Resolution of Functional Diversity in Copper Surface-Associated Marine Biofilms

**DOI:** 10.3389/fmicb.2019.02863

**Published:** 2019-12-11

**Authors:** Yimeng Zhang, Yan Ma, Ruiyong Zhang, Binbin Zhang, Xiaofan Zhai, Wangqiang Li, Liting Xu, Quantong Jiang, Jizhou Duan, Baorong Hou

**Affiliations:** ^1^Key Laboratory of Marine Environmental Corrosion and Biofouling, Institute of Oceanology, Chinese Academy of Sciences, Qingdao, China; ^2^University of Chinese Academy of Sciences, Beijing, China; ^3^Open Studio for Marine Corrosion and Protection, Pilot National Laboratory for Marine Science and Technology (Qingdao), Qingdao, China; ^4^Center for Ocean Mega-Science, Chinese Academy of Sciences, Qingdao, China; ^5^Federal Institute for Geosciences and Natural Resources, Hanover, Germany

**Keywords:** marine, biofilm, biocorrosion, copper-resistance, metal alloy, gene, biofouling

## Abstract

We used metagenomic sequencing combined with morphological and chemical analyses to investigate microbial taxa and functions related to copper-resistance and microbiologically influenced corrosion in mature copper-associated biofilms in coastal seawater for 44 months. Facultative anaerobic microbes such as *Woeseia* sp. were found to be the dominant groups on the copper surface. Genes related to stress response and possible heavy metal transport systems, especially RNA polymerase sigma factors (*rpoE*) and putative ATP-binding cassette (ABC) transport system permease protein (*ABC.CD.P*) were observed to be highly enriched in copper-associated biofilms, while genes encoding DNA-methyltransferase and RNA polymerase subunit were highly enriched in aluminum-associated biofilms and seawater planktonic cells, respectively. Moreover, copper-associated biofilms harbored abundant copper-resistance genes including *cus*, *cop* and *pco*, as well as abundant genes related to extracellular polymeric substances, indicating the presence of diverse copper-resistance patterns. The proportion of *dsr* in copper-associated biofilms, key genes related to sulfide production, was as low as that in aluminum biofilm and seawater, which ruled out the possibility of microbial sulfide-induced copper-corrosion under field conditions. These results may fill knowledge gaps about the *in situ* microbial functions of marine biofilms and their effects on toxic-metal corrosion.

## Introduction

As a prominent mode of microbial life under various natural environments, biofilm consists of cells surrounded by a self-produced matrix of hydrated extracellular polymeric substances (EPS); these components form the 3D architecture of biofilms and contribute to within-biofilm cohesion and surface adhesion (Flemming and Wingender, 2010). In aquatic habitats such as marine environment, surface-associated biofilms are responsible for biofouling or microbiologically influenced corrosion (MIC) of various marine infrastructures (Little et al., 2008; Briand et al., 2017; Chen and Zhang, 2018). Microbes embedded into EPS in biofilms may initially originate from planktonic microflora and will develop into a differentiated community with intercellular communication and interactions with the substratum (Dang and Lovell, 2015). Analyses on surface-associated biofilms revealed that the composition of marine biofilms varies with the substratum type (Briand et al., 2017; Zhang Y. et al., 2019).

Copper and copper alloys are intensively used in marine environment, such as the distribution and piping systems, heat exchangers, offshore structures (Blunn, 1987; Kirk, 1987). These materials are known as toxic and reduce microbial colonization; copper ions accelerate the production of toxic reactive oxygen species (ROS), leading to DNA, lipid, and protein damage in cells, and directly participate in redox reactions with thiols and disulfides to destroy the biological function of proteins with sensitive S groups (Harrison et al., 2007). Despite this, it does not mean that they completely inhibit microbial colonization of copper-tolerant microbes. A previous study has shown that biofilm formation was slower in copper pipes than in plastic pipes, but no difference in microbial numbers was observed between these two materials after 200 days (Lehtola et al., 2004). This finding occurred possibly because the mature biofilms on copper alloys have successfully developed adaptation strategies to survive in toxic conditions after long-term exposure. However, major gaps still exist in our knowledge on these adaptation mechanisms, especially regarding the microbial structural and functional features of mature copper-resistance biofilms after a long-term exposure in seawater.

Understanding of copper-resistance mechanisms is crucial to explore microbial survival strategies and improve biotechnologies such as bio-mining and bioremediation for the removal toxic copper from environments. Hence, in recent years, copper resistance features of microbial communities exposed to copper- polluted natural environments, such as soil (Andreazza et al., 2010; Nunes et al., 2016), coastal seawater (Corcoll et al., 2019), and atmosphere (Meij and Winkel, 2007), have been the subject of intensive research with emphasis on the influence of dispersive copper ions on planktonic microbes. Metallic surfaces in seawater are also useful systems in studying heavy-metal resistance mechanisms. Marine surface-associated biofilms constitute a bank of hidden microbial diversity with functional genes that adapt to toxic metal surfaces, such as transposase gene playing an important role on microbial adaptation to toxic zinc surfaces (Ding et al., 2019; Zhang W. et al., 2019). Therefore, copper-associated biofilms may also harbor special copper-resistance systems.

Recent studies on copper-related MIC in seawater environments focused mostly on the effects of some specific microbial groups on copper alloys, especially the effects of sulfate-reducing bacteria (SRB) (Mansfeld and Little, 1992; Muyzer and Stams, 2008; Chen et al., 2014; Dou et al., 2018). SRB are anaerobic microorganisms that use sulfate as a terminal electron acceptor to degrade organic compounds, leading to the production of corrosive sulfides (Syrett, 1977; Lee and Characklis, 1994; Muyzer and Stams, 2008; Enning and Garrelfs, 2014). The reactions of copper alloys with SRB-produced sulfides contribute majorly to copper-MIC (Mansfeld and Little, 1992; Chen et al., 2014; Dou et al., 2018). In addition, the influence of EPS on corrosion accounts for its capability of binding copper ions (Little et al., 1996) and destroying protective corrosion-product films (Chen and Zhang, 2018). The above results are accomplished under laboratory conditions with the use of cultivable species for biofilm formation. In a complex natural environment, identifying the roles of different microbes in corrosive reactions, and revealing the complex interactions between various functional microbial groups and between microbial communities and materials are difficult. Hence, *in situ* field investigations of biofilms on corroded copper alloys are needed to complement the laboratory studies, by metagenomics that provides valuable information about functional microbes and key genes.

Metagenomic sequencing combined with scanning electron microscopy (SEM) plus energy dispersive X-ray spectroscopy (EDS) was performed on copper-associated biofilms after a long-term exposure in coastal seawater to investigate the microbial functions related to copper-resistance as well as to explore the effect of mature biofilms on copper corrosion in natural marine environment. Biofilms formed on the surface of aluminum alloy and planktonic microbes in surrounding seawater were also investigated for comparisons. The results may have implications in the design of antifouling materials, and the development of new antifouling agents that target specific functional microbes and genes. Moreover, this study provides new insights into the copper-related MIC and copper-resistance mechanisms of microorganisms in natural marine environment.

## Materials and Methods

### Sample Collection

Two commonly used metallic alloys in marine environment, namely, copper alloy (T2) and aluminum alloy (1060), were chosen. T2 primarily comprised copper (wt.% Cu 99.9), and 1060 consisted mostly of Al (wt.% Al 99.60) (Supplementary Table S1). These two alloys were washed with sterile ethanol before being exposed to coastal seawater. All plates were placed vertically and fastened in a frame at a depth of 1–1.5 m below the sea level for 44 months. The sampling sites were located in Hongtang Bay (N 18°17′58′′, E 109°15′18′′), Sanya, Hainan Province, China. This area, with an average temperature of 25.1°C and pH value of 8.5, and belongs to the South China Sea where the salinity is the highest among the four inland seas in China.

Biofilm samples on one side of the alloys were collected and placed in aseptic centrifuge tubes using sterile scrapers. Triplicate biofilm samples from each metallic alloys were collected from three independent plates. The other side of the plates was kept intact and transferred into sterile hermetic bags filled with sampled seawater for further morphological and chemical analysis. Seawater (3 L) samples were filtered through sterile cellulose-ester filter membranes with a pore size of 0.22 μm (diameter 25 mm) to concentrate and collect the microbial biomass. All collected biomass samples were placed on ice and immediately transported to the laboratory. The biofilm and membrane samples were stored at −20°C for DNA extraction within 7 days. The alloy plates were stored at 4°C for morphologic observation within 24 h. The biofilms on the surfaces of copper and aluminum alloy were designated as CuB and AlB, respectively. Seawater samples were abbreviated as SW in the following part.

### Surface and Component Analysis

The surfaces of the two alloys were gently washed by filter-sterilized seawater to remove unattached microbes after the plates were cut into small pieces (no more than 2 cm^2^). Afterward, the biofilms were visualized by SEM (ZEISS ULTRA 55, Germany). Sample preparation for SEM observation was as follows: the surfaces were exposed to 3% (w/v) glutaraldehyde buffered with 0.1 mol/l phosphate buffer for 2 h and dehydrated in gradient ethanol solutions (50, 70, 90, and 100%) for 15 min. The plates were then dried to critical point and coated with gold. After SEM investigation, EDS was used to analyze the chemical components of some targeted regions of the surfaces.

### DNA Extraction and Metagenomic Sequencing

Genomic DNA of biofilm and seawater samples was extracted with a DNeasy PowerSoil Kit (QIAGEN, Hilden, Germany) according to the manufacturer’s instructions. The triplicate DNA extracts with same volumes were finally pooled to meet the requirement of metagenomic sequencing. DNA purity and concentration were checked using a NanoDrop ND-1000UV-Vis spectrophotometer (NanoDrop Technologies, Wilmington, DE, United States) and Qubit dsDNA assay kit in Qubit 2.0 Flurometer (Life Technologies, Foster City, CA, United States), respectively. Agarose gel (1%) electrophoresis was used to assess the integrity of extracted DNA. Sequencing libraries were constructed using NEBNext UltraTM DNA library prep kit for Illumina (NEB, United States) following the manufacturer’s instructions. The size distribution and concentration of purified products in libraries were determined. After the index-coded samples were clustered according to the manufacturer’s recommendations, the libraries were sequenced on the Illumina HiSeq 2500 platform.

### Downstream Data Processing

All metagenomic sequencing reads (raw data) were subjected to Readfq (Version 8.0) to obtain quality-controlled clean data by removing low quality reads (default quality threshold value ≤ 38), the reads containing a certain percentage of N base (default length of 40 bp), and the reads sharing the overlap above a certain portion with adapter (default length of 15 bp). The obtained clean data were then assembled based on the multiple K-mer method (K-mer size values of 49, 55, and 59) using MEGAHIT software (Version 1.0.4-beta) (Qin et al., 2010; Karlsson et al., 2013). The assembled scaffolds were broken from N connection to obtain scaftigs which were then filtered to remove the fragments shorter than 500 bp (Nielsen et al., 2014). Additionally, clean data were mapped to scaftigs of each sample in order to obtain unassembled reads which were then assembled and fragmented to scaftigs again. All obtained scaftigs (≥ 500 bp) were subjected for open reading frame (ORF) prediction to acquire ORFs by MetaGeneMark software (Version 2.10) for further clustering (Karlsson et al., 2013; Li et al., 2014). Clean data were aligned to these gene catalogs using Bowtie (Version 2.24) software. The genes with aligned read number ≤ 2 were removed using CD-HIT software (Version 4.5.8) to obtain the unique genes used for subsequent analysis (Qin et al., 2010). The number of unique genes in each sample was normalized and calculated using transcripts per million reads (TPM) algorithm based on the number of mapped reads and the length of gene to conduct accurate comparisons among different unique genes and among different samples (Qin et al., 2010).

Unique genes were blasted against NCBI-NR (version: 2016-11-15) for taxonomic analyses by using DIAMOND software (Version 0.9.9). For final alignment results of each unigene, lowest common ancestor (LCA) algorithm applied to MEGAN6 software was used to obtain the species annotation results (Huson et al., 2011). After normalization, the relative abundance of an annotated taxonomy was calculated by summing the relative abundance of unique genes annotated to this taxonomy on the basis of LCA annotation and unigene abundance table (Karlsson et al., 2012). To access the accuracy of the taxonomic classification, taxonomic assignments of quality-controlled metagenomic sequencing data to the catalog of marker genes from another mapping tool Metaphlan2 (Truong et al., 2015) were compared.

For functional analyses, the unique genes were blasted against functional databases including Kyoto Encyclopedia of Genes and Genomes (KEGG) database (Version 2018-01^1^) and Carbohydrate-Active Enzymes (CAZy) database (Version 2018-01^2^). The relative abundance of functional genes annotated to KEGG and CAZy database was equal to the sum of the relative abundance of normalized corresponding annotated unigenes. On the basis of functional gene annotation results, unique genes that aligned to Cus, Cop, Pco, and Dsr synthetase gene were retrieved form metagenomes. Taxonomic affiliation of these genes at the genus level was subsequently predicted based on LCA annotation of hits retrieved via blast analysis against NCBI-NR database.

### Data Accession

Metagenomic sequencing data were deposited into NCBI sequence read archive (SRA) under Bioproject accession number PRJNA438021, with Biosample number SAMN11664223–11664228.

## Results

### Biofilm Morphology

**FIGURE 1:**
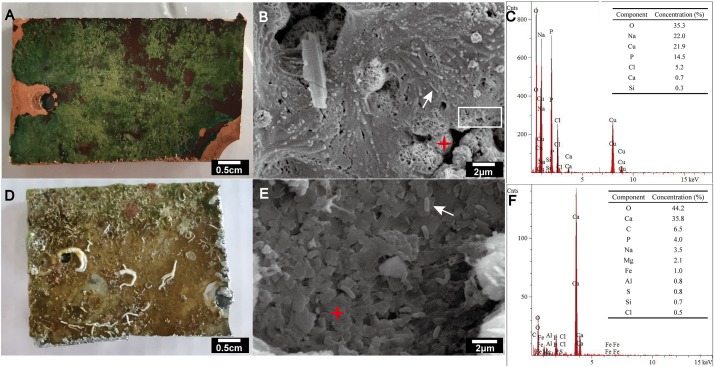
Visual observation **(A,D)**, SEM images **(B,E)** and EDS analysis **(C,F)** of biofilms formed on copper alloy **(A–C)** and aluminum alloy **(D–F)** after exposure to coastal seawater for 44 months. The accelerating voltages used for the SEM of copper **(B)** and aluminum **(E)** alloy were 5,000 and 15,000 V, respectively. Secondary electron imaging was used for all SEM images. The white arrow indicates rod-shaped microbes. The white rectangle indicates the tightly connected region of biofilm and corrosion product. The red asterisk indicates targeting area for EDS analyses.

Scanning electron microscopy and EDS analyses indicated that copper alloy was corroded and the morphology of the marine biofilm formed on copper alloy differed from that of the biofilm formed on aluminum alloy (Figure 1). The biofilm (∼1 mm thick) on the copper alloy was a slimy green layer, covering dark red corrosion products (Figure 1A). SEM micrographs showed a tight connection between the biofilm and corrosion products on the copper surface (Figure 1B). Rod-shaped microbes existed within the biofilms (Figure 1B). The corrosion product of copper alloy was round with multi-porous structure, and primarily comprised copper, oxygen, and chlorine as determined EDS (Figure 1C) in the present and other marine copper corrosion studies (Pope et al., 2000; Chen et al., 2014). By contrast, aluminum alloy developed a loose and uneven colored film (Figure 1D), in which rod-shaped microbes were distributed individually on the surface rather than gathering together (Figure 1E). EDS analysis indicated that the layer below the loose film consisted primarily of calcium and oxygen (Figure 1F).

### Metagenomic Sequencing

As shown in Supplementary Table S2, the metagenomic sequencing of DNA from CuB, AlB, and SW generated 6,629, 6,991, and 6,626 Mb raw data, respectively, which further generated 6,580, 6,882, and 6,565 Mb quality-controlled reads, respectively. The reads above Q30 constituted 93.2, 93.4, 92.0% of those detected in CuB, AlB, and SW samples, respectively. These reads were assembled into 297,836 (CuB), 107,078 (AlB), and 216,353 (SW) scaftigs (≥500 bp) for further gene prediction and analysis. ORF prediction resulted in 489,705 (CuB), 127,817 (AlB), and 322,738 (SW) putative genes. The number of mapped reads at different sequencing depth was shown in Supplementary Figure S1. Rarefication curves of core and pan genes (Supplementary Figure S2) indicated that there were still amount of genes not been captured.

### Microbial Structure

As shown in Table 1, bacteria was the dominant domain on the surface of metallic alloys and seawater based on the results of unique gene annotation, consistent with the results obtained from Metaphlan2 analysis (Supplementary Table S3). Sequencing data also revealed the presence of archaea, eukaryotes and even virus on the surface of metallic alloy. The relative abundance of archaea (0.2%), eukaryotes (0.3%), and virus (1%) in CuB was lower than those in AlB and SW. The abundance of eukaryotes in AlB was higher than that in CuB, and even higher than that in SW.

**TABLE 1:** Relative abundance of microbial kingdom on the metal surfaces and in the seawater based on unique genes assignments to NCBI-NR database.

**Sample**	**Bacteria (%)**	**Archaea (%)**	**Eukaryota (%)**	**Viruses (%)**
CuB	87.6	0.2	0.3	0.1
AlB	51.1	3.5	2.8	1.1
SW	72.1	8.0	1.8	3.5

*CuB, copper alloy-associated biofilm; AlB, aluminum alloy-associated biofilm; SW, seawater.*

**FIGURE 2:**
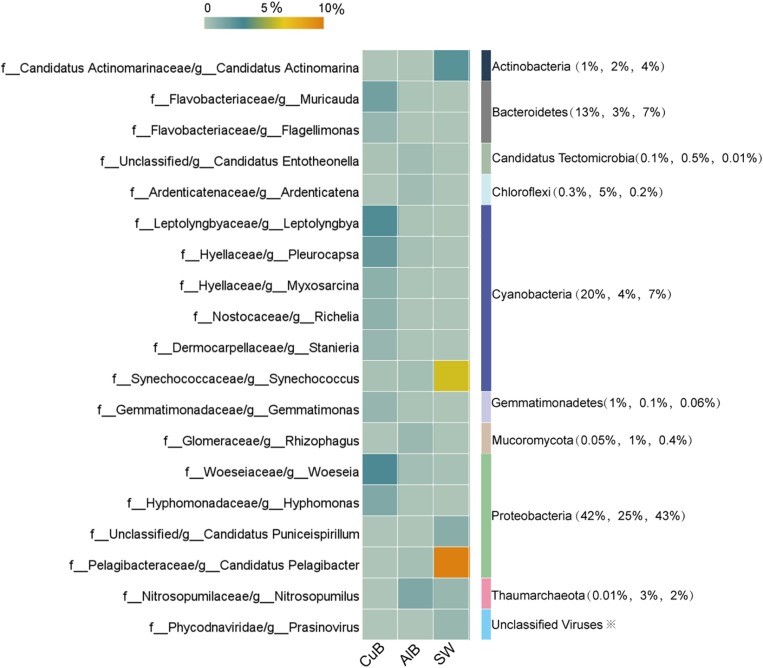
Heatmap of top microbial genera with > 0.5% relative abundance in CuB, AlB, and SW based on unique genes assignments to NCBI-NR database. The X-axis indicates samples and the left Y-axis indicates identified genera. The right column of the Y-axis represents genus-belonging phyla, and the values in brackets indicate the relative abundance of these phyla in CuB, AlB, and SW, respectively. The value of the color scale represents the relative abundance of microbial genera. CuB, copper alloy-associated biofilm; AlB, aluminum alloy-associated biofilm; SW, seawater.

The microbial communities in CuB and SW were dominated by the members of phylum Proteobacteria (42% for CuB, 43% for SW), consistent with the results of Metaphlan2 analysis (Supplementary Table S3). Cyanobacteria (20% for CuB, 7% for SW) was the followed one (Figure 2). For AlB, apart from Proteobacteria (25%) and Cyanobacteria (4%) as the dominant phylum, Chloroflexi accounted for a relatively high proportion (5%). At the genus level (Figure 2), *Woeseia* (3%) representing Proteobacteria, and *Leptolyngbya* (3%) representing Cyanobacteria were the top two most abundant bacterial genera in CuB, followed by *Pleurocapsa* within Cyanobacteria and *Hyphomonas* within Proteobacteria. Different from the predominant genera in CuB, archaeal *Nitrosopumilus* belonging to Thaumarchaeota (2%) was the most abundant genus in AlB. This archaeon has the capacity for chemolithotrophic growth through ammonia oxidation (Walker et al., 2010). Despite similarities in microbial community structure at the phylum level, the dominant planktonic genera in SW and the biofilm ones in metallic alloy exhibit some distinctions. In SW, the dominant Proteobacteria-related genus was *Candidatus Pelagibacter* (SAR11 clade) (10%), followed by Cyanobacteria-related *Synechococcus* (6%), both of which were ubiquitous genera in marine environments. Diverse virus genera including *T4virus* (0.0015%) in CuB, *Bracovirus* (0.0638%) in AlB and *Prasinovirus* (0.75%) in SW, were identified on metallic surfaces (Supplementary Table S4).

### Key Functional Genes

**FIGURE 3:**
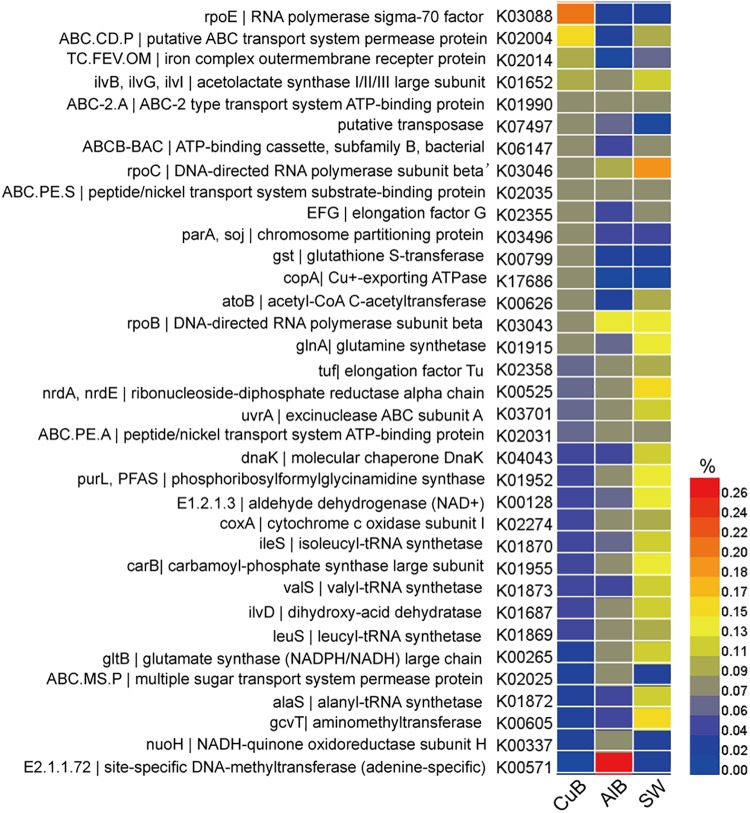
Heatmap of functional genes assigned with KEGG database in CuB, AlB, and SW. The value of the color scale represents the relative abundance of functional genes. CuB, copper alloy-associated biofilm; AlB, aluminum alloy-associated biofilm; SW, seawater.

The functional gene profile of the microbial communities on the metallic surface and in seawater was generated by assigning unigenes against the KEGG database. The relative abundance of top 15 identified genes in each sample, which was calculated based on the reads coverage normalized by the total reads number of the metagenomes. The results are shown in Figure 3. Distinct functional genes were observed for the biofilm samples on metallic surfaces and surrounding seawater samples. Top functional genes annotated from biofilms on metallic alloy (KEGG level 3) were related to membrane transportation belong to environmental information processing, and the genes of planktonic populations in seawater were related to purine metabolism within nucleotide metabolism (Supplementary Table S5). The most abundant functional gene sets were RNA polymerase sigma-70 factor-ecoding gene (*rpoE*) in CuB (0.20%), followed by a putative ABC transport system permease protein (*ABC.CD.P*) (0.16%). A site-specific DNA-methyltransferase (MTase)-encoding gene dominated the aluminum alloy (0.27%). *rpoC*, an ubiquitous but essential gene encoding beta subunit of DNA-directed RNA polymerase complexes for catalyzing RNA synthesis, was the most abundant in seawater sample (0.18%).

### Copper Resistance Genes and Microbes

A wide variety of genes related to copper resistance, including membrane protein- encoding genes *cusABC*, Cu^+^/Cu^2+^-exporting ATPase-ecoding genes *copAB*, copper resistance protein-encoding genes *pcoBCD*, and copper tolerance two-component regulatory system *cusSR* (Table 2) were identified in the metagenomes of CuB. The abundance of the above copper-related genes in CuB metagenomes was higher than that in AlB and SW as shown in Table 2.

**TABLE 2:** Gene count and relative abundance of key functional genes related to copper resistance and sulfide production.

**Gene^a^**	**Description**	**CuB**	**AlB**	**SW**
*cusA*	Cu^+^/Ag^+^ efflux system membrane protein	5333^b^(0.07%)^c^	261(0.003%)	142(0.002%)
*cusB*	Cu^+^/Ag^+^ efflux system membrane fusion protein	2892(0.04%)	141(0.002%)	38(0.0005%)
*cusC*	Multidrug efflux system outer membrane protein	102(0.001%)	60(0.0007%)	–
*cusR*	Copper resistance phosphate regulon response regulator	221(0.003%)	4(0.0001%)	–
*cusS*	Heavy metal sensor histidine kinase	514(0.006%)	51(0.006%)	–
*copA*	Cu^+^-exporting ATPase	6625(0.08%)	1509(0.02%)	1073(0.01%)
*copB*	Cu^2+^-exporting ATPase	4616(0.06%)	546(0.007%)	517(0.006%)
*pcoB*	Copper resistance protein B	2032(0.02%)	169(0.002%)	–
*pcoC*	Copper resistance protein C	110(0.001%)	–	–
*pcoD*	Copper resistance protein D	82(0.001%)	–	–
*dsrA*	Sulfite reductase alpha subunit	44(0.0005%)	98(0.001%)	40(0.0005%)
*dsrB*	Sulfite reductase beta subunit	65(0.0008%)	150(0.002%)	20(0.0002%)

*CuB, copper alloy-associated biofilm; AlB, aluminum alloy-associated biofilm; SW, seawater. ^*a*^The functional genes were annotated against KEGG database. ^*b*^The value indicates the number of annotated genes. ^*c*^The value in the bracket indicates the relative abundance of annotated genes.*

**FIGURE 4:**
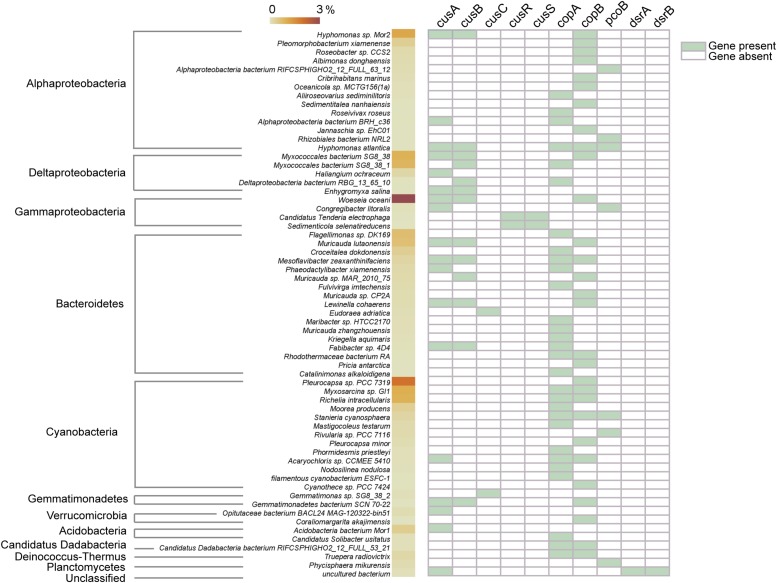
Possible taxonomic assignments of copper-resistance genes. The value of the color scale represents the relative abundance of taxa identified in copper-associated biofilm. The right part containing green and white tiles shows the presence/absence of copper-resistance genes.

The copper-tolerant microbes that possibly contain the above key copper resistance genes in CuB were further explored (Figure 4). In general, most annotated microbes that were predicted to tolerate copper toxicity were affiliated to Proteobacteria (mostly alpha-, delta-, and gamma-Proteobacteria), Bacteroidetes and Cyanobacteria. *Woeseia oceani*, the most abundant taxa identified in CuB, contained two types of membrane proteins CusA and CusB. Additionally, CopB within the family of heavy metal P_1__B_-type ATPases was present in *Woeseia oceani*. Other taxa detected at high levels within Proteobacteria were *Hyphomonas* sp. Mor2 and *Myxococcales* bacterium SG8, which contained at least one type of *cus* and *cop* genes. The results also showed that *Flagellimonas* sp. DK169 and *Muricauda lutaonensis* from Bacteroidetes had the potential to tolerate copper ions. The copper-tolerant taxa affiliated to Cyanobacteria, such as *Pleurocapsa* sp. PCC 7319, *Myxosarcina* sp. Gl1 and *Richelia intracellularis*, usually harbored *cop* genes rather than *cus* genes to resist copper toxicity.

### Key Carbohydrate-Active Enzymes

**FIGURE 5:**
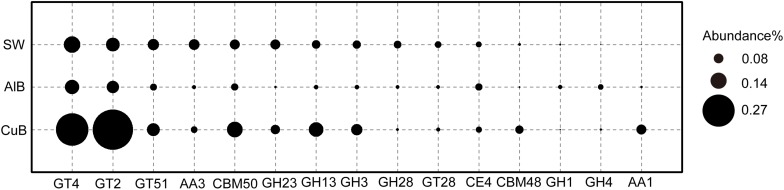
Bubble plot of functional genes encoding CAZy enzymes with ≥ 0.1% relative abundance in CuB, AlB, and SW. The Y-axis represents samples, and the X-axis represents top enzymes encoded by identified functional genes. GT, glycosyl transferase; GH, glycoside hydrolase; CE, carbohydrate esterase; AA, auxiliary activities; CBM, carbohydrate-binding modules; CuB, copper alloy-associated biofilm; AlB, aluminum alloy-associated biofilm; SW, seawater.

Figure 5 shows the top CAZy enzyme-encoding genes identified in three metagenomes. Glycosyltransferases (responsible for formation of glycosidic bonds) (GT2) family 2-encoding gene were the most abundant gene in CuB, whereas GT4-encoding gene was the most abundant in AlB and SW. Compared with AlB and SW, the following were over-represented in CuB: GT4, GT2, glycoside hydrolase (responsible for the hydrolysis and/or rearrangement of glycosidic bonds) family 13 (GH13), carbohydrate-binding model (CBM) family (responsible for adhesion onto carbohydrates) including CBM50 and CBM48, and auxiliary activities family 1 (AA1; redox enzymes that act in conjunction with other CAZy enzymes).

## Discussion

The current study provides the first evidence of the genetic potential of copper-resistance and MIC mechanisms in mature biofilms formed on copper surface in natural marine environments.

### Taxonomic Composition and Function

This study showed that gram-negative, facultative anaerobic microbes dominated copper-associated biofilms, which may be a feature of mature marine biofilms on copper surface with years of immersion. We have previously reported that lactic acid bacteria *Lactobacillus* sp. dominated copper surface after 30-month immersion (Zhang Y. et al., 2019), whereas *Woeseia* sp. dominated after additional immersion in seawater for about 14 months. *Woeseia* genus comprises gram-negative, facultative anaerobic, oxidase-negative and catalase-positive chemoheterotrophic marine microbes (Du et al., 2016). Microbial structures normally change over time even in these mature biofilms, which are affected by external changing environments such as seasonal changes in nutrient loads (Salta et al., 2013). Although the microbial structure in biofilms is dynamic, these results suggest similarities in these mature biofilms among different ages, namely, facultative anaerobic microorganisms dominating the communities. For instance, both of *Lactobacillus* sp. previously detected as dominant taxa (Zhang Y. et al., 2019) and *Woeseia* sp. detected in the present study are facultative anaerobic groups (Das et al., 2016; Du et al., 2016). The facultative respiration mode offers these microbes possibilities to survive under various oxidation–reduction conditions in marine ecosystems. Thus, functional microbial groups with facultative respiration mode can be regarded as typical characteristics of mature copper-biofilms in marine environments.

Microenvironments with various microbial niches in copper-associated biofilms are built by developing a heterogeneous microbial structure. The copper surface is colonized by different types of microbes, such as the facultative anaerobic heterotrophic *Woeseia* sp. (Du et al., 2016) and the aerobic phototrophic *Leptolyngbya* sp. (Shimura et al., 2015). *Hyphomonas* sp. are chemoorganotrophic (Weiner et al., 2000), whereas members of *Leptolyngbya* and *Pleurocapsa* are photoautotrophic and can perform oxygenic photosynthesis (Waterbury and Stanier, 1978; Shimura et al., 2015; Joshi et al., 2018). On one hand, the heterogeneous nature of these biofilms helps microbes survive in the toxic environment. On the other hand, microenvironments with different oxidation–reduction conditions in the biofilm may influence corrosion by forming anode and cathode regions on the copper surface (Keevil, 2004).

The presence of Cyanobacteria (20%) and the striking green color of the biofilm (Figure 1) indicate that phototrophic processes may be important for biofilm development on copper alloy surface. Given the strong ability of coping with changing environments contributed by great metabolic stress responses and physiological active–dormant–active transitions, cyanobacteria often act as pioneers in colonizing bare surfaces (Rossi and Philippis, 2015). The phototrophic activity of Cyanobacteria provide energy and carbon sources from light energy and CO_2_ fixation for themselves, as well as support the growth of other surface colonizers by secreting massive EPS (Rossi and Philippis, 2015). These EPS can further be degraded and be a carbon source for heterotrophic microorganisms associated with the Cyanobacteria (Wong et al., 2015, 2018; Bernstein et al., 2017). Interestingly, the development of copper-associated biofilm is associated with phototrophs, which require more future work to confirm their relationship.

Our results showed that *rpoE* and *ABC.CD.P* that were highly enriched in copper alloy biofilms were also highly enriched in zinc-associated marine biofilms (Ding et al., 2019). By contrast, these two gene sets were in low abundance on aluminum alloy surfaces and seawater (Figure 3). *rpoE* gene, as an important stress-response gene, encodes a significant component of RNA polymerase required for the expression of proteins involved in maintaining the integrity of periplasmic and outer-membrane components (Heimann, 2002). The *rpoE* encoding sigma factor has been proven to be required for full resistance to Zn^2+^ and Cu^2+^ by increasing transcriptome flexibility and producing outer membrane proteins (Egler et al., 2005), therefore it may play significant roles in resisting copper toxicity in copper-associated biofilms. Copper ions, on the other hand, are also vital life element for microbial metabolism due to the redox properties: the copper ions involved in some enzymes help cells to defend against oxidative damage and also serve as electron carriers by alternating between the redox states Cu^+^ and Cu^2+^ (Magnani and Solioz, 2007). Thus another gene related to copper uptake, *ABC.CD.P*, was observed at a high abundance in copper-associated biofilm. This gene encodes ABC transporter permease protein, which relies on periplasmic solute-binding receptors or lipoproteins to recognize and deliver metal ions including zinc and copper ions to the transmembrane transport machinery (Dintilhac et al., 1997; Janulczyk et al., 1999; Claverys, 2001). The high abundance of *rpoE* for stress response and *ABC.CD.P* for possible copper transport may be induced by the high concentration of copper ions in complicated biofilm and in turn help the microbes adjust to heavy-metal stress under field conditions. The dominant taxa identified in copper biofilm, *Woeseia* sp. and the cyanobacteria members including *Leptolyngbya* sp. and *Pleurocapsa* sp., are responsible for these abundant genes based on function and taxonomic analyses. Together with the report of their high abundance in zinc-associated biofilms in marine environments (Ding et al., 2019), this study further confirmed that these two genes played roles in coping with heavy metal stress.

### Copper-Resistance Features

Copper is a vital life element yet inflicts cell damage. Thus, tight control of copper is a cellular necessity, especially under high-copper conditions. Based on KEGG annotation results, except *rpoE* for copper response and *ABC.CD.P* for copper import, microbial communities on the copper surface cope with copper toxicity through efflux pump systems to export toxic copper ions out of cells. This system, identified in the current study, contained two types of efflux pumps to keep the intracellular copper low (Table 2). One type is the ATPase membrane pump that consumes ATP to export copper ions, including CopA and CopB, which encode Cu^+^-exporting ATPase and Cu^2+^-exporting ATPase (Mana-Capelli et al., 2003), respectively. The other efflux pumps are chemiosmotic membrane pumps involving CusABC. CusABC is an enzyme complex that crosses the inner, periplasmic, and outer membrane to bring Cu^+^ out of the cell while taking protons into the cell (Franke et al., 2003). As regulators of CusABC pumps, CusS–CusR two-component systems were also present to up-regulate *cusABC* genes under increased toxic-copper concentrations (Affandi and McEvoy, 2019). In addition to the abovementioned efflux systems, the plasmid-encoded gene *pcoBCD* contained in metagenomes further strengthens the copper-resistance ability by detoxifying copper in the periplasm (Rensing and Grass, 2003).

As expected, the functional species capable of tolerating toxic copper ions dominate the microbiota in copper-affiliated biofilms. Among the dominant taxa, *Woeseia oceani* is found to be possibly affiliated with copper resistance for the first time. The copper-tolerating microbes also show great diversity within Proteobacteria (primarily referring to alpha-, delta-, and gamma-Proteobacteria), Bacteroidetes and Cyanobacteria (Figure 4). Interestingly, the abundant copper-tolerating species within Proteobacteria and Bacteroidetes (e.g., *Woeseia oceani*) generally possess both *cus* and *cop* encoded pumps, whereas Cyanobacteria species contain only *cop* encoded pumps. This finding is consistent with a recent study showing that *cop*-encoded pumps rather than *cus*-encoded pumps are present in Cyanobacteria (Giner-Lamia et al., 2016).

Notably, oxygen availability in copper-associated biofilms may be associated with copper-resistance mechanisms. One of the biochemical pathways for exerting copper toxicity is through accelerating ROS production, which are by-products of Fenton-type reactions under aerobic environments and lead to DNA, lipid, and protein damage (Stohs and Bagchi, 1995). In the aerobic areas of copper-associated biofilm, Cu^+^ is oxidized into Cu^2+^. This means that microbes like cyanobacteria in the outer part of biofilm with high oxygen availability, may be sensitive to Cu^2+^ due to increased ROS production. Cop-efflux systems are primarily used to resist Cu^2+^ in these areas. In the anaerobic part of biofilm, copper toxicity occurs through the destruction of the biological function of proteins by Cu^+^ reactions with sulfur groups in proteins (Harrison et al., 2007). The Cus system identified here, usually worked as an essential Cu^+^ transporter under anaerobic conditions but not essential under aerobic conditions (Outten et al., 2001), is a necessary survival strategy for microbes like *Woeseia oceani* to resist copper in the areas with low oxygen availability.

### EPS and Carbohydrate-Active Enzymes

Extracellular polymeric substances are complex structural components of the biofilms, which include lipopolysaccharides, glycolipids, lipids, proteins, or peptides and nucleic acids (Decho, 2000). The CAZy family produced by microbes in biofilms, taking part in the synthesis and degradation progress of EPS, helps heterotrophic microbes in biofilm obtain carbon sources (Yin et al., 2012). In the present study, some typical genes such as GT2 and GT4-encoding genes were more abundant in CuB than in AlB and SW. GT2 enzymes may be involved in the formation of various polysaccharides including cellulose, chitin, beta-mannan, and alginate. Moreover, GT4 enzymes perform the function of synthesis of sucrose or trehalose (Kadnikov et al., 2013). The identification of high-abundance of genes encoding GT2 and GT4 indicates their potential roles in constructing EPS by producing polysaccharides to form smooth biofilms on copper surface. In addition to GTases, GHases such as GH13 that can degrade polysaccharides with alpha-1, 4-glycosidic bonds into available carbon sources for microbes in biofilms have also identified (Figure 5). Therefore, these surface-associated microbial systems can provide extracellular organic carbon source for microorganisms identified here and in previous studies through EPS synthesis and degradation (Wong et al., 2015, 2018; Bernstein et al., 2017), including Proteobacteria and Cyanobacteria.

The EPS-related genes enriched on copper surface in the present study are also observed in other natural microbial systems exposed to high copper levels, such as microbial mats formed in hypersaline aquatic environments (Wong et al., 2018) and biofilms formed in copper plumbing systems (Keevil, 2004). This finding may be explained by the fact that copper stress in biofilms induces a protective response of microbes through the EPS production (Booth et al., 2011). EPS of biofilms protects cells from copper ions adsorbing to the cell wall by blocking phosphoryl groups that would bind the copper ions (Gonzalez et al., 2010), and by chelating copper ions within EPS (Harrison et al., 2005). Consequently, the entry of copper ions into the cells is prevented. Compared with copper-associated biofilm, the film formed on aluminum alloy seemed more like monolayer rather than biofilm based on the distribution patterns of individual cells and low EPS-related genes. The calcium carbonate deposits identified on the surface of aluminum alloy would negatively influence EPS production and biofilm formation (López-Moreno et al., 2014). From this aspect, higher EPS production may be a feature of copper-associated biofilm in marine environments.

### Biofilm-Related MIC of Copper Alloy

As shown in most reported studies on the MIC of copper alloys in artificial seawater, microbiologically produced sulfides (produced mostly by SRB) elicit the most attention and are regarded as the primary cause of MIC (Mansfeld and Little, 1992; Gouda et al., 1993; Chen et al., 2014). However, *dsrA* and *dsrB*, key functional genes encoding sulfite reductase that participate in the last step of dissimilatory sulfate reduction to generate sulfides, were found in very low abundance on corroded copper surface even at the same order of magnitude as aluminum alloy and seawater (Table 2). Commonly studied SRB groups were also absent. Together with the non-detection of metal sulfide on copper surface by EDS analysis (Figure 1C), we suggest that the MIC of copper caused by SRB produced sulfide has been overstated in the past. Conversely, the identification of an unclassified uncultured bacterium with both *dsr* and copper resistance gene *cusA* in CuB suggests that uncultured bacteria may also be a potential threat causing copper corrosion and thus requires consideration.

Based on the above results, we inferred that the MIC of copper alloys (T2) may be accelerated mostly by the heterogeneity of biofilms attributed to the heterogeneous microbial structure, as well as by the binding capacity for ions and the destruction of EPS protective films in natural marine environments. On one hand, heterogeneous microbial structure primarily formed by aerobic and facultative anaerobic microbes leads to the creation of oxygen gradients due to oxygen/nutrient consumption and pH gradient formation (Picioreanu and van Loosdrecht, 2002). Thus, corrosion zones containing cathodic and anodic areas induced by different concentrations of dissolved oxygen (DO) appear in the biofilms, where metallic copper is oxidized to copper ions in anodic areas (low DO concentrations) and dissolved oxygen is reduced in cathodic areas (high DO concentrations) (Vargas et al., 2017). The non-detection of key hydrogen cycling genes including hydrogenase (*ech*) and dehydrogenase (*coo*) encoding genes suggests that microbes in copper-associated biofilms fail to accelerate cathodic reactions by consuming hydrogen, which is usually observed in SRB-mediated steel corrosion (Lee and Characklis, 1993; Angell et al., 1995). On the other hand, higher production of EPS in biofilms leads to the destruction of protective copper-oxide film, thereby promoting corrosion (Chen and Zhang, 2018). EPS can also stimulate the anodic dissolution of metallic copper by chelating copper ions (Little et al., 1996). These conceptual explanations may provide guidance to substantial studies on copper corrosion.

## Conclusion

Metagenomic analysis suggests that marine surface-associated biofilms formed on the copper-alloy surfaces possess a more distinct microbial and functional gene composition compared with those formed on aluminum alloy and planktonic microflora. Facultative anaerobic microbes that can resist toxic cupric ions dominate mature copper-associated biofilms with years of formation. This study is the first to show that the two predominant functional genes identified in copper-associated biofilms, stress response *rpoE* gene and possible copper-transporter *ABC.CD.P* gene, are significant to microbial communities in natural formed biofilms to control heavy metal homeostasis. These two genes coexist with *cus* and *cop*-encoding copper-efflux systems, implying that the mature biofilms have the great genetic potential to tolerate copper ions. Diverse and overrepresented CAZy-encoding genes are also special characteristics of copper-associated biofilm. These genes influence the synthesis and degradation of EPS and further affect biofilm heterogeneity and copper-resistance ability. Therefore, copper alloy surfaces immersed in natural seawater may be significant sources of new functional potential and vital environments to explore heavy metal resistance mechanisms.

The present study further shows that field studies on marine biofilms formed on corroded materials using omics-approaches can complement laboratory investigations on MIC caused by a single species or its biofilms. SRB-produced sulfide does not significantly contribute to copper-related MIC in natural marine environments, suggesting that future laboratory studies on copper-MIC should focus more on microbial heterogeneity and EPS. It is also noteworthy that the reliability of conclusions made by metagenomic sequencing is positively related to the number of samples. Hence, to further understand copper resistance and MIC mechanisms, more biofilm samples collected from various types of copper alloys and sea areas with different environmental parameters are needed in the future.

## Data Availability Statement

The datasets generated for this study can be found in the NCBI sequence read archive (SRA) under Bioproject accession number PRJNA438021, with Biosample numbers SAMN 11664223, 11664224, 11664228.

## Author Contributions

YZ was responsible for designing and conducting the experiments, analyzing the data, and drafting the manuscript. YM and RZ revised the manuscript and provided much needed insight into results interpretation. BZ, XZ, WL, LX, and QJ took part in plate placement and sample collection. JD as the corresponding author and BH have overseen all aspects of this project in terms of scientific significance.

## Conflict of Interest

The authors declare that the research was conducted in the absence of any commercial or financial relationships that could be construed as a potential conflict of interest.

## Footnotes

1 http://www.kegg.jp/

2 http://www.cazy.org/
