# Supplementary material for: Metagenomic Resolution of Functional Diversity in Copper Surface-Associated Marine Biofilms
Source: Front Microbiol. 2019 Dec 11;10:2863. doi: 10.3389/fmicb.2019.02863 (PMC6917582; doi:10.3389/fmicb.2019.02863)
Supplement: Supplementary file 1 [file Table_1.docx]

Metagenomic resolution of functional diversity in copper surface-associated marine biofilms

Yimeng Zhang ^1,2,3,4^, Yan Ma^1,3,4^, Ruiyong Zhang ^5^, Binbin Zhang ^1,3,4^, Xiaofan Zhai ^1,3,4^, Wangqiang Li^1,3,4^, Liting Xu^1,3,4^, Quantong Jiang ^1,3,4^, Jizhou Duan ^1, 3, 4*^, Baorong Hou^1,3,4^

^1^Key Laboratory of Marine Environmental Corrosion and Biofouling, Institute of Oceanology, Chinese Academy of Sciences, Qingdao, China

^2^University of Chinese Academy of Sciences, Beijing, China

^3^Open Studio for Marine Corrosion and Protection, Pilot National Laboratory for Marine Science and Technology (Qingdao), Qingdao, China

^4^Center for Ocean Mega-Science, Chinese Academy of Sciences, Qingdao, China

^5^Federal Institute for Geosciences and Natural Resources, Hanover, Germany

*** Correspondence:**Pro. Jizhou Duan
duanjz@qdio.ac.cn

Table S1 The proportion of major component of metallic alloys

| Component | Copper alloy (T2) (wt. %) | Aluminum alloy (1060) (wt. %) |
| --- | --- | --- |
| Cu | 99.9 | ≤0.05 |
| S | 0.005 | - |
| Pb | 0.005 | - |
| Fe | 0.005 | 0~0.4 |
| Sb | 0.002 | - |
| As | 0.002 | - |
| Bi | 0.001 | - |
| Al | - | 99.6 |
| Si | - | 0.25 |
| Mg | - | ≤0.05 |
| Zn | - | ≤0.05 |
| Mn | - | ≤0.05 |
| Ti | - | ≤0.03 |
| V | - | ≤0.05 |

Table S2 Features of the metagenomic sequencing data of biofilm and seawater samples.

| Sequencing parameter | CuB | AlB | SW |
| --- | --- | --- | --- |
| Raw data size (Mb) | 6 629 | 6 991 | 6 626 |
| Data size of quality-controlled reads (Mb) | 6 580 | 6 882 | 6 565 |
| Percentage of reads with precision>Q30 (%) ^a^ | 93.2 | 93.4 | 92.0 |
| GC content (%) | 53.3 | 49.9 | 47.2 |
| Number of assembled scaftigs (>=500 bp) | 297 836 | 107 078 | 216 353 |
| Average length of scaftigs (bp) | 1095 | 842 | 929 |
| Max length of length of scaftigs (bp) | 91 345 | 28 158 | 34 950 |
| N50 length (bp) ^b^ | 1160 | 774 | 925 |
| Number of ORFs ^c^ | 489 705 | 127 817 | 322 738 |

Abbreviations: CuB, copper alloy-associated biofilm; AlB, aluminum alloy-associated biofilm; SW, seawater.

a Q30 indicates the sequencing error rate is below 0.001.

b N50 indicates the length of the scaftigs reaching to 50% of the total length of scaftigs.

c ORF: Open Reading Frame, indicating the number of predicted genes.

Table S3 Relative abundance of top annotated phylum using Metaphlan2

| Kindom | Phylum | CuB (%) | AlB (%) | SW (%) |
| --- | --- | --- | --- | --- |
| Bacteria | Proteobacteria  Firmicutes  Actinobacteria  Cyanobacteria  Marinimicrobia | 42  29  3  0  0 | 18  3  1  2  0.03 | 89  0.02  0  0.1  8 |
| Archaea | Thaumarchaeota | 0 | 76 | 2 |

Abbreviations: CuB, copper alloy-associated biofilm; AlB, aluminum alloy-associated biofilm; SW, seawater.

Table S4 Relative abundance of top 5 virus identified at the genus level in copper alloy, aluminum alloy and seawater.

| Genera | CuB(%) | AlB(%) | SW(%) |
| --- | --- | --- | --- |
| *T4virus* | 0.0015 | 0.0417 | 0.0873 |
| *Schizot4virus* | 0.0010 | 0.0002 | 0.0019 |
| *Prasinovirus* | 0.0007 | 0.0052 | 0.7548 |
| *Cytomegalovirus* | 0.0007 | 0.0002 | 0.0001 |
| *Alphabaculovirus* | 0.0005 | 0.0341 | 0.0013 |
| *Bracovirus* | 0.0003 | 0.0638 | 0.0034 |
| *Yuavirus* | 0.0005 | 0.0095 | 0.0003 |
| *Lymphocystivirus* | - | 0.0082 | 0.0013 |
| *Chlorovirus* | - | 0.0052 | 0.0074 |
| *Coccolithovirus* | 0.0002 | 0.0004 | 0.0047 |
| *Cafeteriavirus* | - | 0.0003 | 0.0038 |

Abbreviations: CuB, copper alloy-associated biofilm; AlB, aluminum alloy-associated biofilm; SW, seawater.

Table S5 Annotated gene functions against KEGG database

| Annotated function (level 3) | Annotated function (level 2/level 1) | CuB(%) | | AlB(%) | SW(%) |
| --- | --- | --- | --- | --- | --- |
| ABC transporters | Membrane transport/Environmental Information Processing | 1.21 | 1.27 | | 1.42 |
| Purine metabolism | Nucleotide metabolism/Metabolism | 1.16 | 1.15 | | 1.88 |
| Two-component system | Signal transduction/Environmental Information Processing | 1.07 | 0.45 | | 0.79 |
| Pyrimidine metabolism | Nucleotide metabolism/Metabolism | 0.93 | 0.87 | | 1.54 |
| Oxidative phosphorylation | Energy metabolism/Metabolism | 0.86 | 1.02 | | 1.23 |
| Quorum sensing | Cellular community/Cellular Processes | 0.79 | 0.76 | | 0.98 |
| Pyruvate metabolism | Carbohydrate metabolism/Metabolism | 0.75 | 0.60 | | 1.09 |
| Glyoxylate and dicarboxylate metabolism | Carbohydrate metabolism/Metabolism | 0.74 | 0.71 | | 1.05 |
| Ribosome | Translation/Genetic Information Processing | 0.71 | 0.78 | | 1.26 |
| Aminoacyl-tRNA biosynthesis | Translation/Genetic Information Processing | 0.58 | 0.76 | | 1.19 |
| Glycine, serine and threonine metabolism | Amino acid metabolism/Metabolism | 0.67 | 0.71 | | 1.20 |

Abbreviations: CuB, copper alloy-associated biofilm; AlB, aluminum alloy-associated biofilm; SW, seawater.


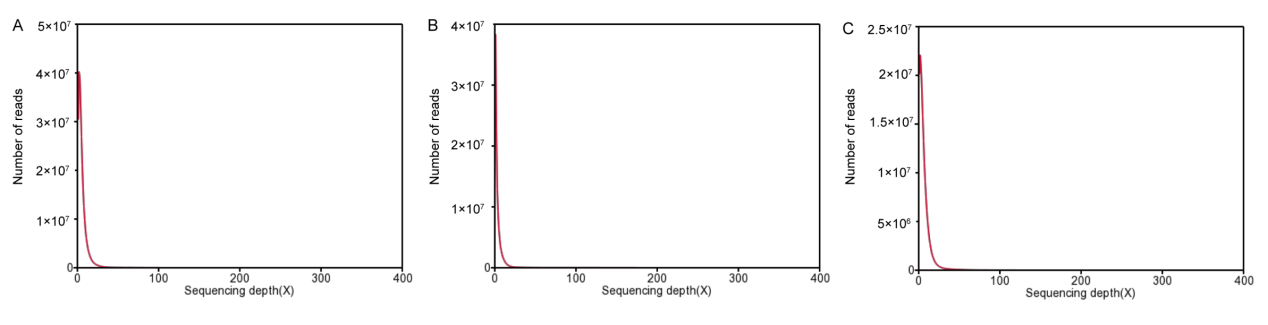


Figure S1 Sequencing depth and the number of corresponding mapped reads for CuB (A), AlB (B) and SW (C). CuB, copper alloy-associated biofilm; AlB, aluminum alloy-associated biofilm; SW, seawater.


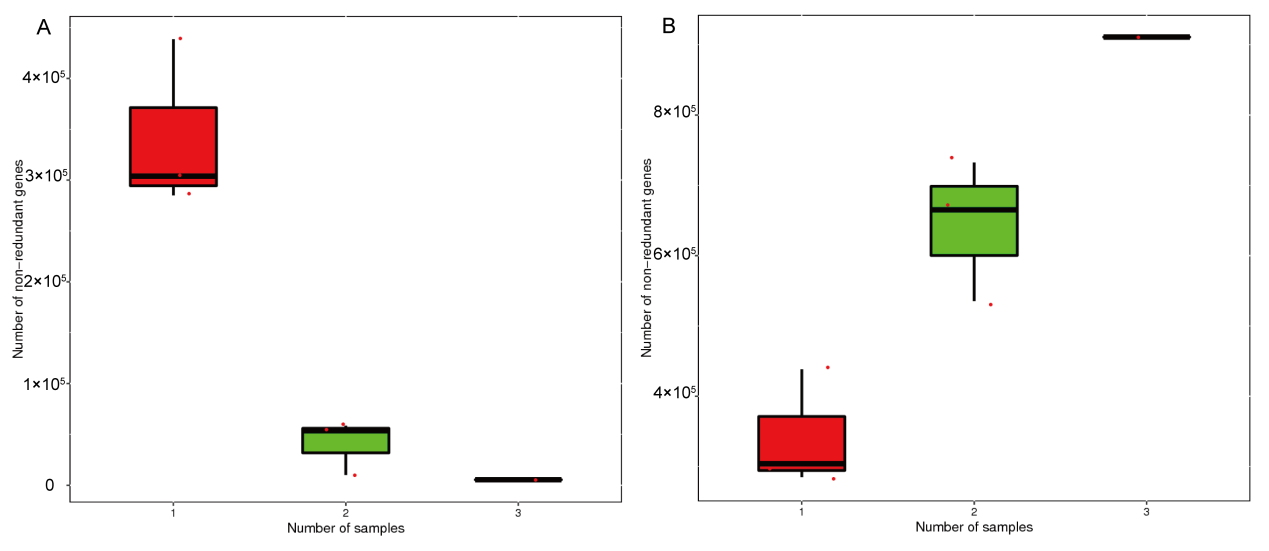


Figure S2 Rarefaction curves of core (A) and pan (B) genes.
